# Predicting the natural history of metabolic syndrome with a Markov-system dynamic model: a novel approach

**DOI:** 10.1186/s12874-021-01456-x

**Published:** 2021-11-27

**Authors:** Abbas Rezaianzadeh, Esmaeil Khedmati Morasae, Davood Khalili, Mozhgan Seif, Ehsan Bahramali, Fereidoun Azizi, Pezhman Bagheri

**Affiliations:** 1 Colorectal Research Center, Shiraz University of Medical Sciences, Shiraz, Iran; 2 Center for Circular Economy, Business School, University of Exeter, Exeter, UK; 3 Prevention of Metabolic Disorders Research Center, Research Institute for Endocrine Sciences, Shahid Beheshti University of Medical Sciences, Tehran, Iran; 4 Department of Epidemiology, School of Health, Shiraz University of Medical Sciences, Shiraz, Iran; 5 Noncommunicable Diseases Research Center, Fasa University of Medical Sciences, Fasa, Iran; 6 Endocrine Research Center, Research Institute for Endocrine Sciences, Shahid Beheshti University of Medical Sciences, Tehran, Iran; 7 Shiraz University of Medical Sciences, Shiraz, Iran

**Keywords:** Metabolic syndrome, Markov-system dynamics, Natural history

## Abstract

**Background:**

Markov system dynamic (MSD) model has rarely been used in medical studies. The aim of this study was to evaluate the performance of MSD model in prediction of metabolic syndrome (MetS) natural history.

**Methods:**

Data gathered by Tehran Lipid & Glucose Study (TLGS) over a 16-year period from a cohort of 12,882 people was used to conduct the analyses. First, transition probabilities (TPs) between 12 components of MetS by Markov as well as control and failure rates of relevant interventions were calculated. Then, the risk of developing each component by 2036 was predicted once by a Markov model and then by a MSD model. Finally, the two models were validated and compared to assess their performance and advantages by using mean differences, mean SE of matrices, fit of the graphs, and Kolmogorov-Smirnov two-sample test as well as R^2^ index as model fitting index.

**Results:**

Both Markov and MSD models were shown to be adequate for prediction of MetS trends. But the MSD model predictions were closer to the real trends when comparing the output graphs. The MSD model was also, comparatively speaking, more successful in the assessment of mean differences (less overestimation) and SE of the general matrix. Moreover, the Kolmogorov-Smirnov two-sample showed that the MSD model produced equal distributions of real and predicted samples (*p* = 0.808 for MSD model and *p* = 0.023 for Markov model). Finally, R^2^ for the MSD model was higher than Markov model (73% for the Markov model and 85% for the MSD model).

**Conclusion:**

The MSD model showed a more realistic natural history than the Markov model which highlights the importance of paying attention to this method in therapeutic and preventive procedures.

**Supplementary Information:**

The online version contains supplementary material available at 10.1186/s12874-021-01456-x.

## Introduction

The study of natural history of chronic diseases is doubly complex due to their complex nature and multifactorial causality [1–3]. Because of this complexity, there are few detailed descriptions about chronic diseases natural history [4]. The aim of a study on natural history is to clarify the factors that affect the overall risk of transition from one stage to another as a diseases progresses (or regresses) [5]. Among the existing studies, some have looked at the natural history of diseases and their pathophysiology from a systems biology and complex and dynamic systems perspective [6]. Other studies, on the other hand, have illustrated the natural histories with complex statistical methods [7–10]. The most common methods for investigation of dynamic and complex situations and their progression are simulation-based statistical methods. Among these, Markov models, which pay special attention to random changes in processes (stochastic processes), are more important. Markov and system dynamics models clearly belong to two different scientific fields, but similar to the system dynamics models, the Markov models provide a powerful framework for analyzing dynamic systems [11–13]. However, Markov model requires a lot of computational capacity when a system becomes complex due to increase in the number of states and transitions. The MSD model is a hybrid model that combines Markov and system dynamics approaches to overcome the limitations of Markov models in modeling complex systems. Indeed, despite the difference between the Markov and the system dynamic models in terms of the stochastic and deterministic nature of the states, due to the important similarity of Markov model with system dynamic model in terms of “state” and “transition”, these two models can be combined with each other or even in some cases converted to each other [14]. This hybrid model have been mainly used in non-medical fields and repairable systems for reliable analysis in a more realistic way [12, 15–17]. In fact, in a MSD model the failure and repair or control rate of a system, which are time-varying indexes, are considered for transient availability modeling or analysis of system reliability in calculations [15, 18]. Due to information feedback theory, easiness of tweaking parameters to test different hypotheses and possibility of providing solutions to various problems by adjusting flow rates [16], system dynamics models are a suitable solution to eliminate computational limitations of Markov models.

Metabolic Syndrome (MetS) is a global public health challenge with a plethora of increasing research around its epidemiology and physiological mechanisms [19–24]. However, there are few studies on its natural history which have provided contradictory findings [7–10, 25–27]. MetS is a very complicated disorder and one can have a different combination of the syndrome components at any given time, depending on their lifestyle. To be precise, one can be in one of the states of “no component”, “isolated hypertension”, “isolated overweight/obesity”, “isolated hyperglycemia”, “isolated dyslipidemia”, “obesity + hypertension”, “obesity + dyslipidemia”, “obesity + hyperglycemia”, “hypertension + dyslipidemia”, “hypertension + hyperglycemia”, “dyslipidemia + hyperglycemia”, and a set of combinations of three or four components [28, 29]. The MetS is quite dynamic and one can transit from one state to another. This dynamicity of the disorder development in individuals makes it a proper candidate for an MSD approach. Therefore, this study was conducted to evaluate the performance of a MSD model in a context of investigating MetS natural history in a large population-based study.

## Methods

### Study type and participants

This retrospective study was undertaken on 4 waves of Tehran Lipid and Glucose Study (TLGS), ranging from year 1999 to year 2016 [30–32]. Data collection and measurement procedures, sampling processes, eligibility criteria for participants, and definitions of MetS criteria in TLGS are published elsewhere [33, 34].

The aim of the study was to evaluate the performance of a MSD model in investigation of MetS natural history (transition between 4 components of MetS, i.e. abdominal obesity, hypertension, hyperglycemia, high triglycerides with low HDL (dyslipidemia) and their combinations (12 states)). To be precise, the investigation encompassed calculating and predicting transition probabilities (TPs) between the mentioned 12 states over a period of 21 years (2015–2036) through a compartmental MSD model. The findings then were compared with those of a Markov model to see which model worked better.

### Markov model

**Fig. 1:**
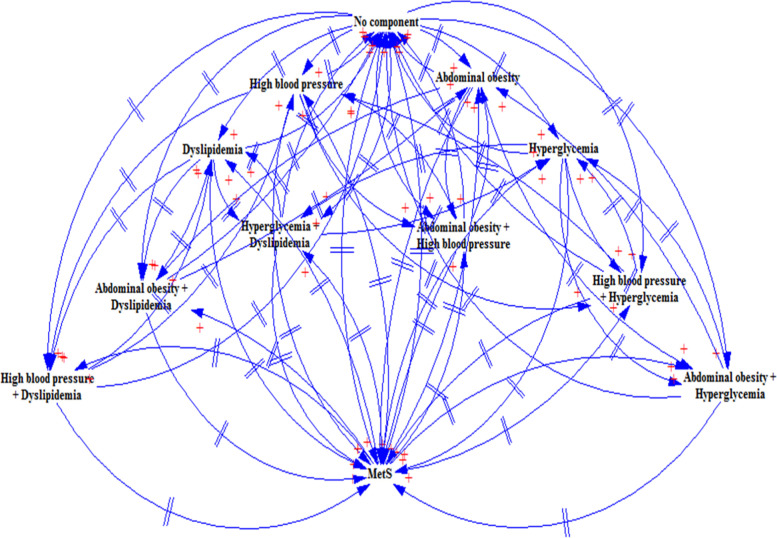
A 12-State dynamic transition diagram for MetS natural history

At the beginning of this section, a 12-state Markov model was designed and used to describe the natural history of MetS (Fig. 1). A Markov process is a random model for describing a sequence of probable events in which the probability of each event depends only on the present time, not preceding event. In other words, if the status of a process is known at times x_1_, x_2_…x_n_, then it can be said that only the latest information (that is the state of the process at the x_n_ time), is sufficient to predict the future progression of the process (X_n + 1_). Accordingly, the Markov dependency that is introduces as Markov properties (Memoryless) is assumed as follow:

$$P\left({X}_{n+1}={x}_{n+1}|{X}_1={x}_1,{X}_2={x}_2,\dots, {X}_n={x}_n\right)=P\left({X}_{n+1}={x}_{n+1}|{X}_n={x}_n\right)$$

Of course, it should be noted that the Markov dependency to the current state, can also be of a different order than the first, e.g., of the second-order [35]. So, in many practical situations the first-order dependency is sufficient but not always justifiable. However, this is a fundamental assumption in the Markov model which is mainly considered. Markov process can be fully described by its TP function or p_ij_ (t) which is the probability that a system is in (j) state at time (t), provided that the process starts from time (t = 0) and state (i). Hence when (i) = x _(n-1)_ [36], then one can write a Markov process as follow:

$${P}_{ij}\ \left({X}_{n+1}= sj|{X}_n= si\right)=P\left({X}_n= sj|{X}_{n-1}= si\right)$$

That, (s) is the total number of states that a system can occupy at any given time. In our model, the time criteria for calculating the TPs in each phase across all 12 states was triennial and the final matrix of TPs was equal to the average of total values of TPs in all periods [9]. Also, the final state (i.e. MetS), was considered as an absorbing state. An absorbing state is a state in which no transition to any subsequent state will take place [37].

Based on the number of states in our model, a 12 × 12 transition matrix was used to calculate the TPs.

$$\mathrm{P}=\left[\begin{array}{ccc}{P}_{11}& \cdots & {P}_{112}\\ {}\vdots & \ddots & \vdots \\ {}{P}_{121}& \cdots & {P}_{1212}\end{array}\right]$$

### MSD model design

In order to design a MSD model, control rate (CR) and failure rate (FR) indices were first calculated (section A in Additional file 1). FR and CR indices are used to evaluate the reliability of system models. They are also used to evaluate the effects of various interventions in a system model (SD). The interventions in our study were medicinal (i.e. self-reported consumption of different medications to control blood pressure, blood lipids, and blood sugar levels) and lifestyle-based interventions (i.e. TLGS phase II to reduce risk factors for non-communicable diseases in some participants [38]). A SD model performs the risk prediction process by using these two indices in a pre-fabricated model that is the product of actions-reactions between the states in a Markov model. FR (CR) indicates any progress (regress) from less (more) components towards more (less) components across the natural history of MetS.

In the CR calculation, both for lifestyle interventions and medicinal therapies, patients who were on the MetS state were not included in the calculation. Also, since no medicinal intervention was needed for healthy individuals, people with no-components state were not included in the CR calculation for medicinal interventions. Mean values of CR and FR were considered as the final values.

**Fig. 2:**
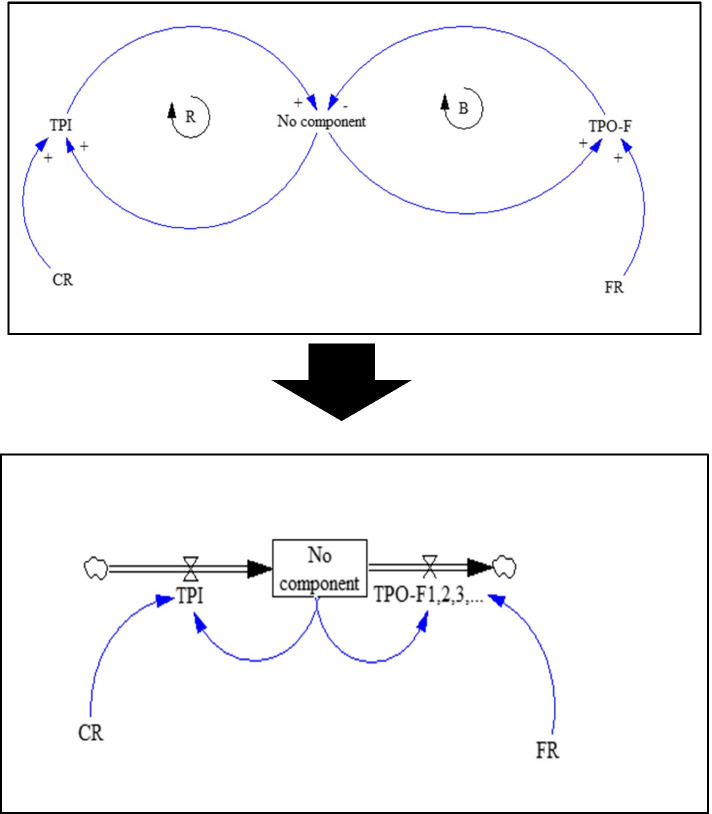
Causal loop and stock-flow diagrams for the no-component state

**Fig. 3:**
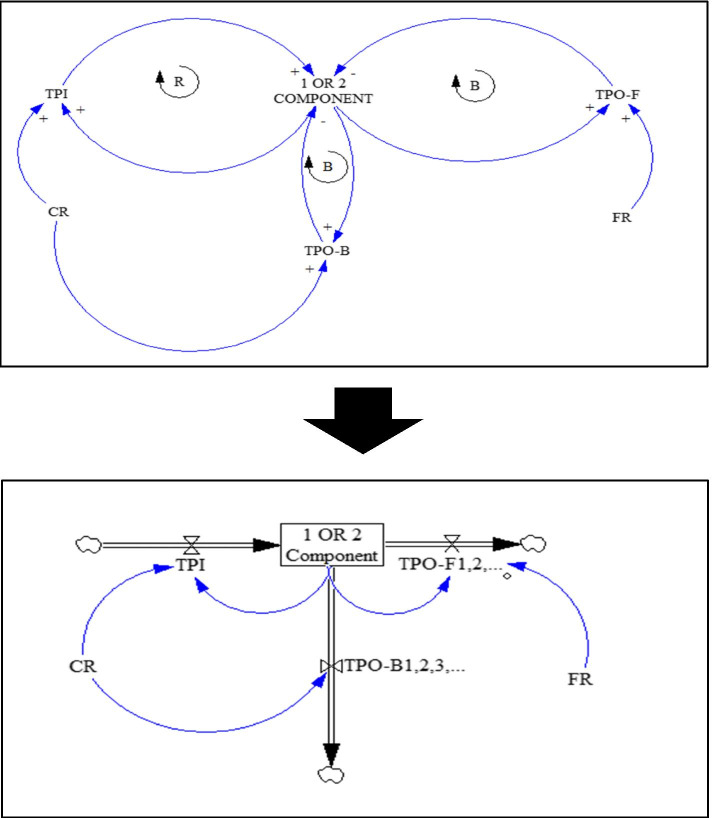
Causal loop and stock-flow diagrams for 1 and 2-component states

**Fig. 4:**
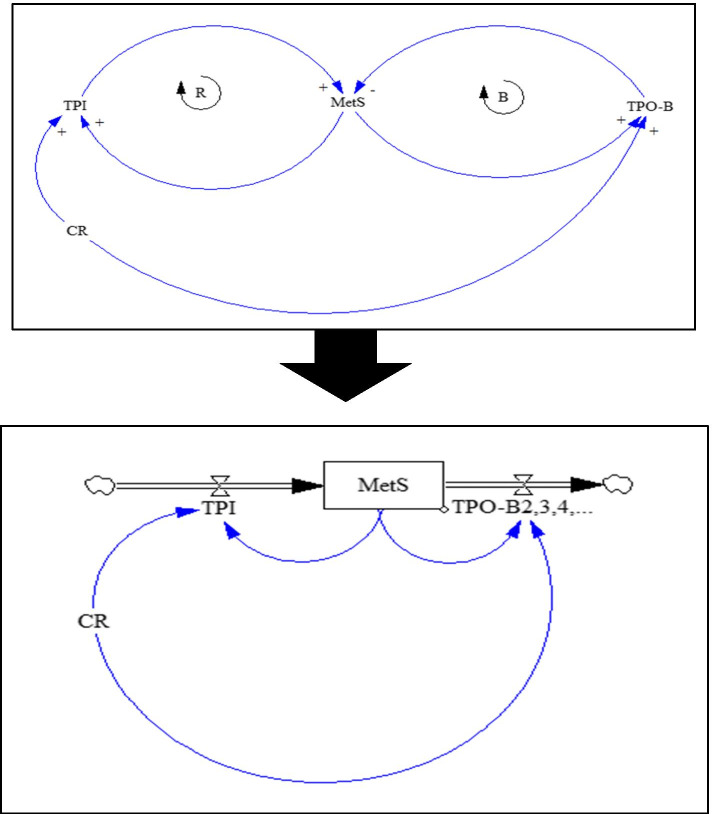
Causal loop and stock-flow diagrams for MetS state

After calculating the CR and FR indices, based on our Markov diagram (Fig.1), causal loop and stock and flow diagrams were drawn for formulation of the SD model separately for no component, 1-component, 2-component and MetS (Figs. 2, 3 and 4). In fact, at this stage, in order to perform simulations, qualitative models (causal loop diagram) were transformed into quantitative models (stock and flow diagram).

In these SD diagrams, the dynamic processes of transitions from each component to the preceding component (i.e. recovery or transition probability backward (TPO-B)), to the next component (i.e. disease progression or transition probability forward (TPO-F)), and lack of transition (stoppage) are shown as in-transition probabilities (TPIs) under the influences of CR and FR. B sign in the diagrams is indicative of a balancing cycle and R sign refers to a reinforcement cycle. To all of these transitions, which are in fact longitudinal (vertical) transitions, the types of lateral (horizontal) transitions must be added. Lateral transitions (Width TP = TPW) are the conversion (replacing) of each of the 1-components to other 1-components and also each of the 2-components to the other 2-components, which include a total of 21 transitions (6 transitions in 1-component and 15 in 2-component). Therefore, lateral transitions were defined in the form of TPW index, which is not affected by CR and FR. The final MSD model, which has become a quite complicated model of MetS and its components, is shown in Additional file 1 (refer to Fig. 1 in Section C of the Additional file 1).

### Statistical analysis

#### Predictions with Markov model

In a Markov model, ***P***_***ij***_^(***n***)^ is the probability of transition from state (i) to state (j) in n^th^ step. To calculate the matrix p^n^ (the transition matrix of step (n)), one must multiply the matrix (P) n times by itself, which the (P_ij_) element in the matrix p^n^ will be the same of P_ij_^(n)^ [39]. For Risk prediction, the time horizon, based on the minimum average time of transition of individuals directly from no component to the MetS (which was approximately 2 years) in the follow-up periods, for 7 periods (3-year) (2015–2036) calculated and presented (refer to Section B of the Additional file 1). Given that transition probabilities were non-homogeneous or time dependent in our model, instead of transition probability, transition rate was used in which transitions are calculated as per unit time or instantaneous, which is equivalent to rate. Hence, the predictive rate was used in the Y axis as proportion of individuals who developed the MetS from various states over time period per total person-time.

#### Markov model validation

To validate Markov model performance, values of parameters in the fourth wave of the TLGS were predicted using the data from three preceding waves. The predicted values then were compared with the actual data using measures of mean differences and mean standard error of matrices. As a visual evaluation, a graph of the proportionality of the predicted data values with the empirical data in terms of trends was drawn in general.

### Verification and prediction with the final MSD model

In order to validate the MSD model, the same validation steps in the case of the Markov model were repeated. Finally, for risk prediction by continuing the existing conditions such as Markov model, Time horizon, for 7 periods (3-year) (2015–2036) was calculated and presented. Since the SD model is actually a differential equations system whose its order depends on the number of variables, in the risk prediction section, to calculate the value (N) of each state, the following differential equation was designed. As an example, the equation is designed for the no component state and applies to other states as well.

$$\mathrm{No}\ \mathrm{component}\ \left(\mathrm{nc}\right)\ \mathrm{equation}=\frac{d\ (nc)}{d\ (t)}=\left({\upalpha}_{\mathrm{inflow}\ \left(\mathrm{t}0\right)}\times {\mathrm{nc}}_{\left(\mathrm{t}0\right)}\right)-\left({\upalpha}_{\mathrm{outflow}\ \left(\mathrm{t}\mathrm{n}\right)}\times {\mathrm{nc}}_{\left(\mathrm{t}\mathrm{n}\right)}\right)$$

On the other hand, based on the above differential equation, the integral equation based on the 3-year interval was written as follow:

$$nc={\int}_{\mathrm{t}0}^{\mathrm{t}3}\left[\left(\mathrm{CR}\times \mathrm{TP}\times {\sum}_{\mathrm{N}}\mathrm{n}\mathrm{c}\left(\mathrm{t}0\right)\right)+\left(\sum \mathrm{CR}\times \mathrm{TP}\times {\sum}_{\mathrm{N}}\mathrm{otherstonc}\left(\mathrm{t}\mathrm{n}\right)\right)-\right(\mathrm{FR}\times \mathrm{TP}\times {\sum}_{\mathrm{N}}\mathrm{n}\mathrm{c}\mathrm{toothers}\left(\mathrm{t}\mathrm{n}\right)\Big]$$

Finally, the integral equation for calculating the values of TPO-F and TPO-B, which are longitudinal transitions (from no component to MetS), and TPI, which are considered as insider transitions and are described in the previous sections, are as follows:

$$TPO-F=\int \left[{N}_{\left( origin\ state\right)}\times {TP}_{\left( origin\ \mathrm{state}\ \mathrm{to}\ \mathrm{next}\ \mathrm{state}\right)}\times {FR}_{\left( origin\ \mathrm{state}\right)}\right]$$

$$TPO-B\ and\ TPI=\int \left[{N}_{\left( origin\ state\right)}\times {TP}_{\left( origin\ \mathrm{state}\ \mathrm{to}\ \mathrm{next}\ \mathrm{state}\right)}\times {CR}_{\left( origin\ \mathrm{state}\right)}\right]$$

The integral equation of lateral transitions was also written as follows:

$$TPW=\int \left[{N}_{\left( origin\ state\right)}\times {TP}_{\left( origin\ \mathrm{state}\ \mathrm{to}\ \mathrm{next}\ \mathrm{state}\right)}\right]$$

#### Evaluation of models’ performance

A triple approach was used to compare the performance of Markov and MSD models. At first, mean standard error of matrices, mean of the differences, and fit of the graphs were used to compare the two models’ outputs. Then, using Kolmogorov-Smirnov two-sample test, closeness of predicted and empirical (goodness of fit) samples distributions in both Markov and MSD models was compared. Finally, in the third approach, the value of the R^2^ index was calculated based on a simple linear regression model between the actual and predicted values. As a result, the model with a smaller mean SE, a smaller mean difference, a more appropriate graph, and also a higher goodness of fit as well as higher R^2^ was selected as the desired one. Also, for quantification of uncertainty in predicted model’s performance assessment in both models, standard errors for estimated transitions (predictive rates) as a measure of the accuracy of the resulting estimates that provide ability to objectively assess the quality of the reported estimates, was calculated. To estimation of the standard error associated with each transition, our approach was to use a bootstrap method [40] with 1000 iterations and combine results.

### Additional analysis

Mean and percentage were used for descriptive analysis of baseline and follow-up waves of the TLGS. Also, Cochran’s Q test was used to examine the significance of revealed trends in data. 0.05 was set as the significant value. IBM SPSS Statistics software for Windows version 24 (IBM Corp, Armonk, NY), excel 2016, and R-4.0.3 (“msm [41]“and “markovchain [42]“packages) were used for data analyses. The maximum likelihood method was used for parameter estimation in methods that have been implemented within “markovchain” and “msm” packages.

### Ethical considerations

As this study was conducted on the TLGS data, it is ethically subject to the ethical considerations observed in the TLGS project. The study was also ethically approved by National Committee of Ethics in Iranian Biomedical Research (code# IR.SUMS.REC.1398.835).

## Results

### Demographic variables description

56.16% (7235) of participants in TLGS sample (12,882) were female. At baseline, the mean of participants’ age was 31.34 ± 17.3 years (median age = 29 years).

### States description

Table 1 shows the status and trend of changes in 12 states of MetS during study periods. In general, the highest prevalence in baseline belonged to isolated dyslipidemia. In terms of difference between baseline and final stage values, states of “no component”, “isolated dyslipidemia”, “obesity + dyslipidemia”, “hypertension + dyslipidemia”, and “dyslipidemia + hyperglycemia” all had decreasing trends and the highest decrease was related to “hypertension + dyslipidemia”. Other combinatorial states had increasing trends and the highest increase belonged to “obesity + hyperglycemia” state.

**Table 1:** Longitudinal change of MetS states among participants over the study period

States of MetS	Baseline		F1		F2		F3		F4		Change (%)	P*
	Count	%	Count	%	Count	%	Count	%	Count	%		
No Component	1604	12.5	672	5.2	917	7.1	1154	9.0	1248	9.7	− 22.1	< 0.0001
Isolated Overweight/Obesity	1360	10.6	927	7.2	1370	10.6	2462	19.1	2725	21.2	100.3	
Isolated Hypertension	106	.8	38	.3	72	.6	67	.5	139	1.1	31.1	
Isolated Dyslipidemia	3902	30.3	2872	22.3	2112	16.4	1037	8.0	705	5.5	− 81.9	
Isolated Hyperglycemia	143	1.1	96	.7	279	2.2	303	2.4	456	3.5	218.8	
Obesity + Hypertension	233	1.8	128	1.0	131	1.0	284	2.2	508	3.9	118.0	
Obesity + Dyslipidemia	3325	25.8	4864	37.8	3767	29.2	3002	23.3	2017	15.7	−39.3	
Obesity + Hyperglycemia	190	1.5	183	1.4	379	2.9	978	7.6	1192	9.3	527.3	
Hypertension + Dyslipidemia	264	2.0	190	1.5	178	1.4	94	.7	97	.8	− 63.2	
Hypertension + Hyperglycemia	26	.2	22	.2	79	.6	50	.4	118	.9	353.8	
Dyslipidemia + Hyperglycemia	350	2.7	538	4.2	737	5.7	351	2.7	333	2.6	−4.8	
MS	1379	10.7	2352	18.3	2861	22.2	3100	24.1	3344	26.0	142.4	

^*^Two-sided *p*-value significance level = 0.05, and Cochrane test

### TPs values

The overall TPs matrix is given in Table 2. Over the study period (4 follow-up periods), probability of direct and non-stop transition from “no component” to MetS was 8.6%. The highest transition probability from “no component” to other states belonged to “isolated abdominal obesity”. Among isolated components, the highest TP towards MetS was related to hyperglycemia and hypertension, respectively. Among the composite components, the highest TP towards MetS belonged to obesity & hypertension which was the highest value of TP toward MetS among all components and their combinations (41.1%), which was generally introduced as the main initiator. The diagonal row in the matrix (Table 2) indicates the probability of remaining in same state, no transition, over time. In overall, people with MetS had the highest probability (60.2%) of no transition over time.

**Table 2:**
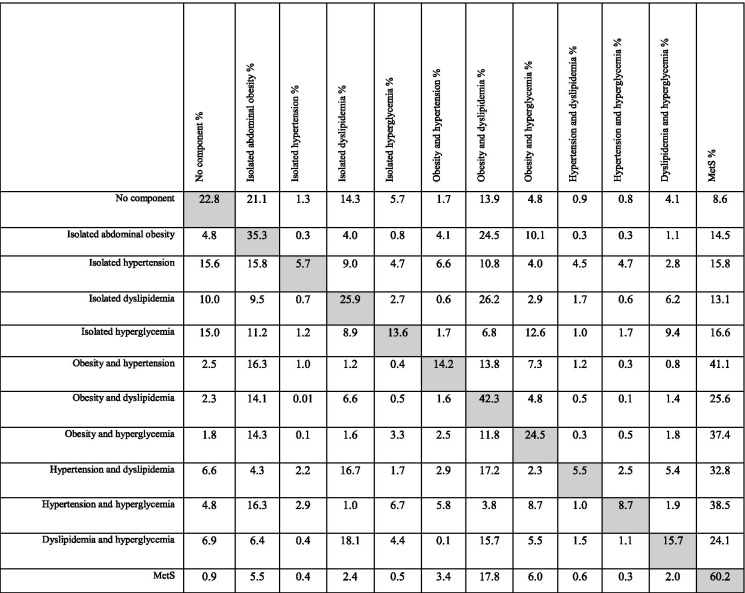
Matrix of transition probabilities (%)

### Markov model

**Fig. 5:**
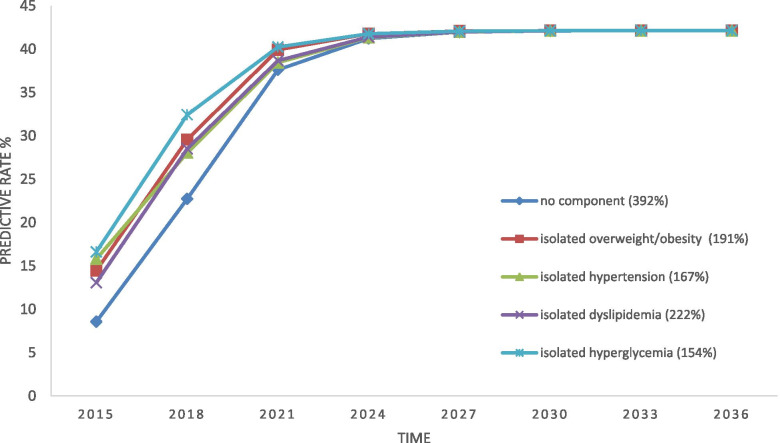
Risk of progression towards MetS for isolated components in Markov model

Markov predictions show that as the time continues over the years, the probability of transition towards MetS for all isolated states would first experience an upward trend until the sixth year, and then all the states would have a same probability of transition. Among the isolated states, people in “no component” state will experience the highest increase in the upward trend towards MetS and those with hyperglycemia will experience the least increase, before reaching to a constant probability (steady state) (Fig. 5). In other words, the highest rate of MetS seems to occur among people with no component state. At the same time, the highest rate of progression toward the MetS was related to hyperglycemia and the lowest was associated with no component.

**Fig. 6:**
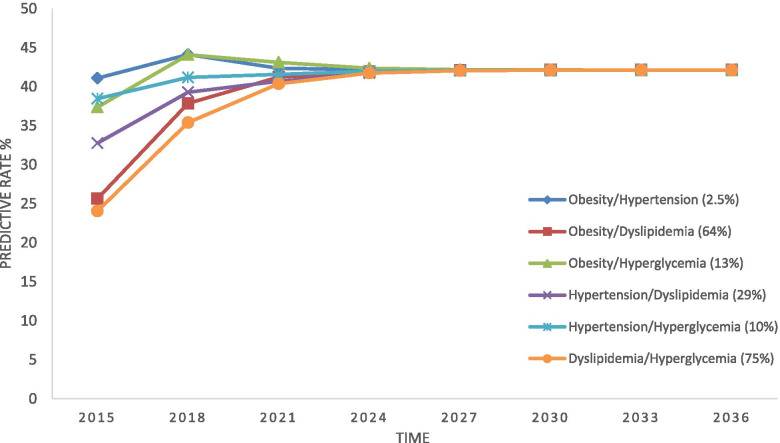
Risk of progression towards MetS for composite components in Markov model

In the case of composite states, the predictions showed that except for “obesity + hypertension” and “obesity & hyperglycemia” which would have a decreasing or a constant trend of transition towards MetS, other states would all first have an increasing trend for 6 years and then would flat-out. Before reaching to the steady probability, the highest progression toward the MetS was associated with obesity & hyperglycemia, and the highest progression was associated with dyslipidemia & hyperglycemia (Fig. 6). In other words, the highest rate of MetS among all the composite states, until reaching to the steady level, seems to occur in people with “dyslipidemia + hyperglycemia”.

### Validation of the Markov model

In general, the mean of differences was 0.0562 and the mean SE of the predicted matrix from the actual matrix for the fourth period of TLGS was 0.003684. Also, the trend analysis showed that the fit between values in empirical and predicted data was favorable. Moreover, in terms of closeness of values, with an overestimation of about 5.62%, the estimated values were relatively desirable (Figs. 2 and 3 in section C Additional file 1). Generally, the evaluation was suggestive of relative adequacy of the Markov model in risk prediction.

### MSD model

The overall CR and FR indices are presented in Table 3 (detailed tables can be found in Additional file 1 section D). The SD model was built to examine the progression of each component towards the MetS (separately for isolated components and composite components). For this purpose, the CR and FR values along with transition probabilities were entered into the final MSD model (Fig. 1 in Additional file 1 section C) and a risk prediction process was simulated.

**Table 3:** Control and failure rates in metabolic syndrome interventions

		No component	1-component	2-component	MetS
Overall	CR	36.66	62.29	77.37	43.94
	FR	63.34	37.70	22.63	–

**Fig. 7:**
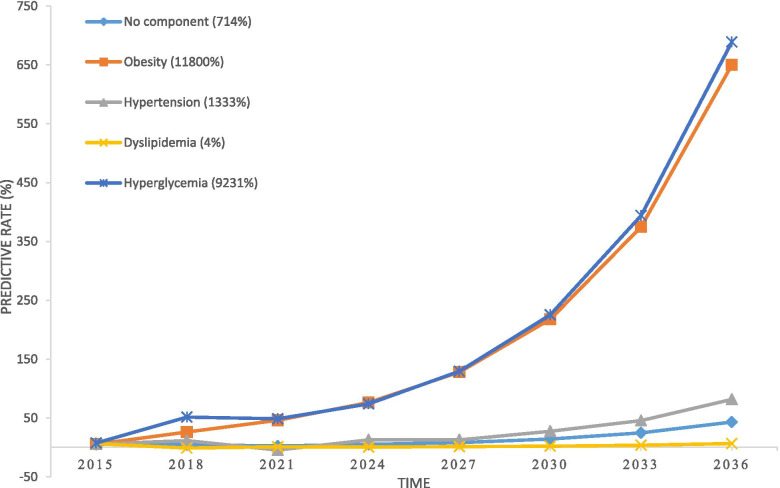
Risk of progression towards MetS for isolated components in MSD model

**Fig. 8:**
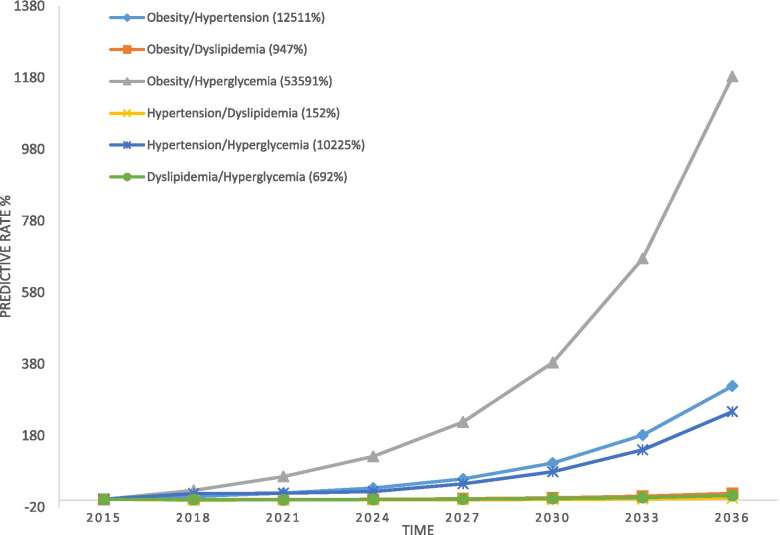
Risk of progression towards MetS for composite components in MSD model

According to the MSD modeling outputs, among the isolated components, the highest progression rate towards MetS was related to hyperglycemia and obesity, respectively. The trends of other components had also a small upward slope and the lowest rate of progression belonged to dyslipidemia (Fig. 7). In the case of composite components, the rate of “obesity + hyperglycemia” progression towards MetS was higher than others composites (Fig. 8). But, in overall, progression slope of composites was greater than that of isolated components (except for obesity and hyperglycemia). The lowest progression slope was related to “hypertension + dyslipidemia”.

### Validation of the MSD model for risk prediction

The mean difference between the predicted values and the empirical values was 0.04911 and the mean SE of the predicted matrix from the real matrix in the fourth period of TLGS was 0.002056. Also, the trend analysis showed that the fit between values in empirical and predicted data was desirable. Moreover, in terms of proximity of values, with an overestimation of about 4.9%, the estimated values were desirable (Figs. 4 and 5 in section C of Additional file 1). Overall, the evaluation indicated that the MSD model performance, in terms of risk prediction, was satisfactory.

### Evaluation of models’ performance

According to the evaluation outputs, both the Markov and MSD models were shown to be desirable models for risk prediction. But, according to greater proximity of predictions made by the MSD model to the real (empirical) conditions (i.e. fitter graphs of values proportionality, lower mean difference (less overestimation), lower SE of the general matrix, and also significance of the MSD model test (*p* = 0.808 for MSD model and *p* = 0.023 for Markov model) which is indicative of equal distribution of real and predicted samples in the MSD model) and Finally, a higher R^2^ for the MSD model (73% for the Markov model and 85% for the MSD model), the MSD model was shown to be a more desirable model for predictions. Also, uncertainty quantifications given in section E in Additional file 1.

## Discussion

In this study, a MSD model was designed to model the natural history of MetS, i.e. progression from its components. The model then was compared with a Markov model to evaluate their performance. The findings showed that both the Markov and MSD models were adequate enough to predict the secular trends of the MetS. But based on the greater proximity of the predictions made by the MSD model to the real data gathered in TLGS, the MSD model was introduced as the desirable model.

The MSD model has a systemic approach and adopts a comprehensive and integrated view to the processes that lead up to MetS. For instance, a MSD model enriches one’s understanding of the natural history of MetS by integration of the effectiveness of MetS-driven therapeutic and life-style interventions (i.e. control and failure rates) into the model. It also enriches the understanding by being open and inclusive to dynamicity of MetS components and the fact that one can shuffle back and forth between simple and complex components of the MetS over time. Therefore, the authors thought that a MSD model is a good match to sheer complexity of the MetS and might outperform other models, e.g. Markov model, in terms of risk predictions.

The risk prediction by Markov model in our study showed that all states/components first showed an upward trend towards MetS until the ninth year. Then, all the trends levels off at a same risk value. This pattern of trends (only the trends and not the time until leveling-off) was seen in other studies [7–10]. However, in the risk prediction process with the MSD model, which, there was no similar evidence, assuming that the existing conditions continued, the progression of all states toward the MetS (with differences between various states in different conditions) was upward which was completely different from the process observed in Markov modeling, both in our study and in other studies [7–10]. To be specific, the predictions made by the MSD model is a component-specific prediction that is not comparable with the general trends reported in other studies. In fact, in other studies, the general trend of MetS is drawn and described, while in our study, the trend of each components is described as part of the natural history of the disease. In other word, the trends shown by Markov model mainly refers to progression of the disease as a whole and lifetime, but the MSD model reveals the progression and dynamicity of each component in the natural history towards MetS. To clarify it more, it seems that since the Markov model does not systemically consider the natural history of MetS, interactions between components over time, non-linear knock-on effects of changes in each component on other components, and influence of external factors (e.g. interventions) on the natural history of the MetS are not considered in the modelling. As a result, rather than seeing the natural history of progress of components and states as a whole, the natural history of each component or state is examined and predicted separately. In this case, the real contribution of each component or state in the occurrence of MetS and its trend is probably not seen in full, and the rate of progress and change in progress are not accurately calculated. Importantly, in this study, our aim was to model the development trend of MetS components as different compounds as the natural history of MetS, rather than the development of MetS over time as a whole, that usually can be seen in other studies. Clinically and mechanically, as seen in this study, the highest rate of progression has been from no component to isolated components and from isolated components to composite components and finally to MetS, which illustrate a cumulative and ascending process over time.

In addition to Markov model, there are a few other prospective risk prediction models that have been used to predict the process of MetS development. For example, one study used a biomarker-base model [43] and the risk of MetS based on age and gender was predicted for 5 to 10 years into future. In another study, Framingham Risk Score (FRS) model was used [44] and an upward trend and an irregular trend was predicted for high and low risk people, respectively. Retrospective studies have also been another way to investigate the developmental process of MetS. For instance, it was shown that overall prevalence of MetS over a 15-year period had an upward trend, but incidence of MetS from different components had an irregular and relatively upward trend among children & adolescents [33] and adults [45]. The differences between our findings and those of others in this section are probably related to different study methodologies.

Accordingly, lack of similar studies in terms of methodology was a challenge for our study. In fact, although we showed that MSD outperformed Markov model (and probably other models) in revealing the developmental process of MetS, but unless it is widely used in various medical fields, a clear-cut judgment on functional advantages and strength of MSD model would be avoided. This is where we cordially invite researchers to work on in future.

## Conclusion

The natural history of many chronic diseases, e.g. MetS, develops through multistate and dynamic paths. This chronicity, state-multiplicity, and dynamicity therefore calls for systemic approaches in order to understand and control these health problems. In this study, a MSD model showed to outperform a commonly used Markov model in revealing the developmental process of MetS over time. Our findings, therefore, invite researchers to adopt MSD models in investigation of chronic and complex health problems and test its practicalities.

## Supplementary Information

**Additional file 1.**

## Data Availability

The datasets used and/or analyzed during the current study are available from the corresponding author on reasonable request.
