# Supplementary material for: Predicting the natural history of metabolic syndrome with a Markov-system dynamic model: a novel approach
Source: BMC Med Res Methodol. 2021 Nov 27;21:260. doi: 10.1186/s12874-021-01456-x (PMC8627615; doi:10.1186/s12874-021-01456-x)
Supplement: Supplementary file 1 — Additional file 1. [file 12874_2021_1456_MOESM1_ESM.docx]

**Appendix:**

**Section A:**

MSD models consist of 4 stages (42, 43):

1- Drawing the State Transition Diagram as the input of the MSD model 2- Data Collection step to calculate the state transition diagram as well as failure rate (FR) and control rate (CR) indices 3- Drawing the Comprehensive MSD Simulation Model (Building the Comprehensive MSD Simulation Model) which actually results from converting state transition diagrams of the system to rate and level diagrams. 4- And finally MSD Simulation or the main simulation of the MSD model that was developed in the previous step. This step actually uses the transition probabilities calculated in the Markov stage along with the FR and CR indices to simulate and model the system. By using these indicators and formulating the natural history of system progress, a comprehensive MSD model is obtained.

**Section B**

A random process $X=\left[X_{t},t\in N\right]$ is called a Markov chain, if the following condition holds for any $t\in N$and $j\in E$ that in which E is a countable set:

$$P\left[X_{t+1}=j\left|X_{0},X_{1},\ldots,X_{t}\right.\right]=P\left[X_{t+1}=j\left|X_{t}\right.\right]$$

The above expression indicates the probability of the existence of a system at time (t + 1) in state (j), provided that, at time (t) the state of the system and the states of the previous events are known.

Markov continuous process is fully described by its transition probability function or p_ij_ (t) which is the probability that a system is in state (j) at time t, provided that the process starts at time t = 0 in state (i). Hence when (i) = x (0) (15):


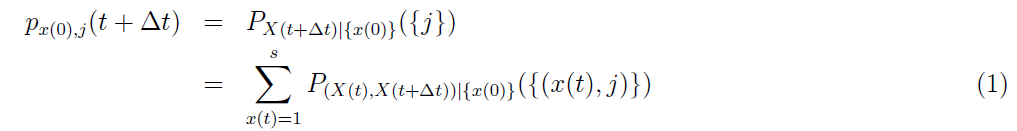


In this equation, (s) is the total number of states that a system can occupy at any given time.

Using the general law of probability, Equation 1 can be written as follows:


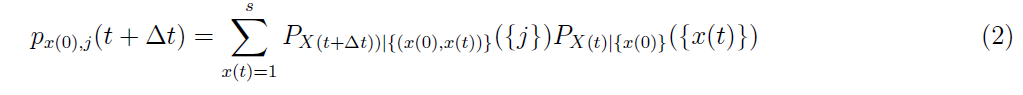


The memoryless property of the Markov model results in:


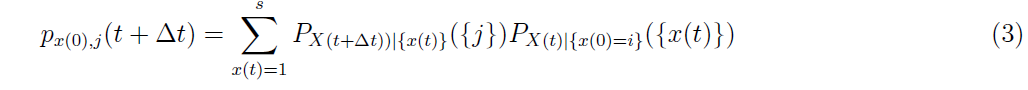


Which is defined as follows:


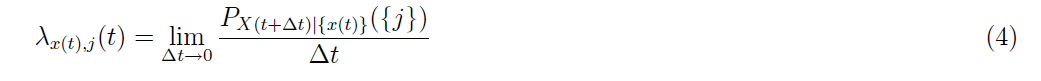


and considering:


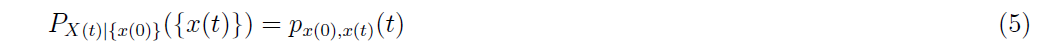


will have:


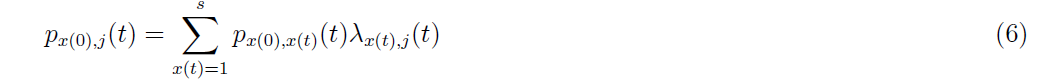


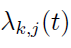
This equation is known as the Chapman Kolmogorov equation equation.

In this equation, the quantity is equal to the transition rate from state (k) to state (j) at time (t). In general, the transition rate value can be time-varying or even state-dependent, but this value is usually assumed to be constant, known as the stationarity assumption. In some sources, this mode is also called the homogeneous (or stationary) Markov model. There are two main assumption in the Markov chain model: 1) Only the last state affects the next state, known as the Markov property. This assumption is also known as the Time-dependent performance measures default. 2) The time stationarity property assumption, which states that transition probabilities do not depend on time which the transition occurs. This assumption is also known as the Time-independent performance measures assumption (44).

According to Equation 6, the continuous time Markov model is obtained by the following equation:


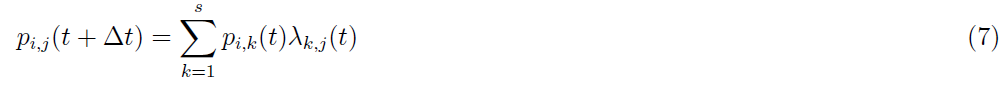


In this equation, the values of transition probabilities p_ij_ (t) are a continuous function of time with the following properties for all characters (i), (j) and (t):


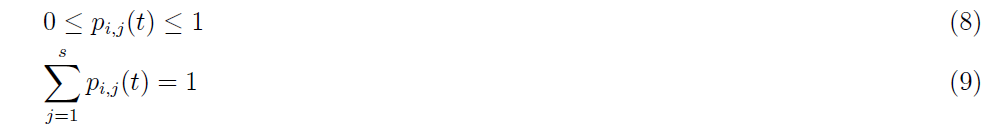


The quantity of transfer rates also has the following characteristics:


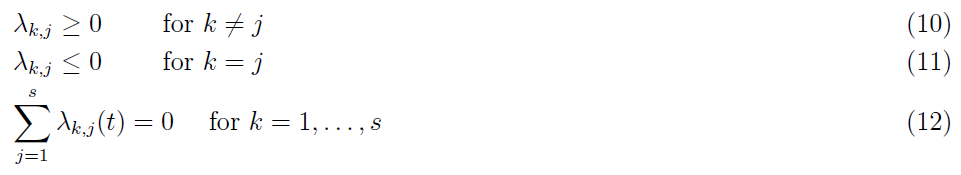


The instantaneous rate of change of state probabilities is obtained from the following formula:


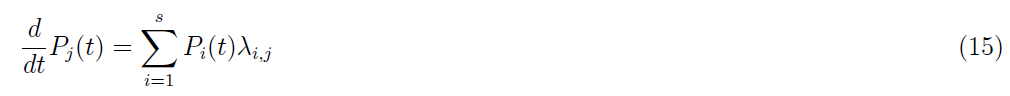


**Section C:**

Figure 1)
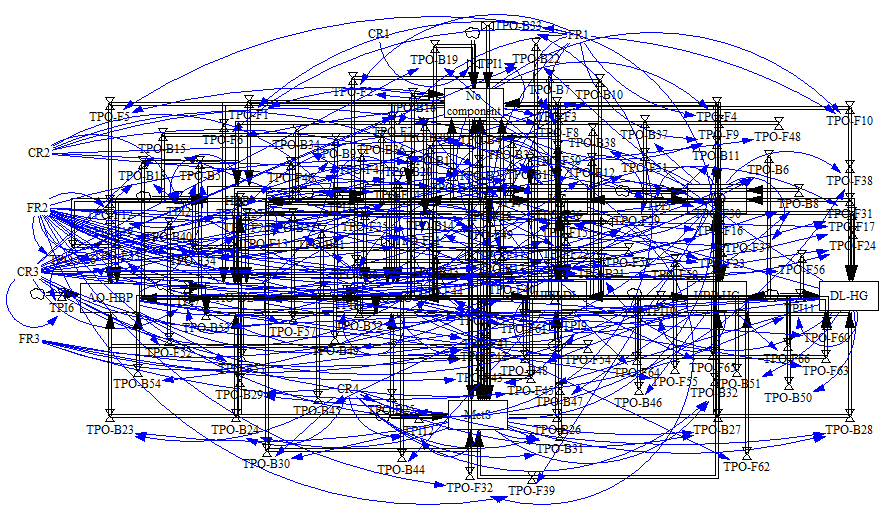
The final system dynamic model of MetS

Figure 2) fitting status between empirical and predicted values for isolated components

Figure 3) fitting status between empirical and predicted values for composite components

Figure 4) fitting status between empirical and predicted values for isolated components

Figure 5) fitting status between empirical and predicted values for isolated components

**Section D:**

Overall:

For lifestyle-centered intervention (total of 4 periods):

|  | No component | 1-com | 2-com | MS | total | Failure rate | Control rate |
| --- | --- | --- | --- | --- | --- | --- | --- |
| No component | 224 | 253 | 114 | 20 | 611 | 63.34 | 36.66 |
| 1-com | 272 | 1245 | 871 | 171 | 2559 | 40.72 | 59.28 |
| 2-com | 87 | 730 | 1478 | 578 | 2873 | 20.12 | 79.88 |
| MS | 11 | 116 | 387 | 751 | 1265 | - | 40.63 |

For drug intervention (total of 4 periods):

|  | No component | 1-com | 2-com | MS | total | Failure rate | Control rate |
| --- | --- | --- | --- | --- | --- | --- | --- |
| 1-com | 135 | 219 | 136 | 52 | 542 | 34.69 | 65.31 |
| 2-com | 188 | 712 | 908 | 607 | 2415 | 25.14 | 74.86 |
| MS | 66 | 550 | 1408 | 2258 | 4282 | - | 47.26 |

| Overall | | |
| --- | --- | --- |
|  | Failure rate | Control rate |
| No component | 63.34 | 36.66 |
| 1-com | 37.705 | 62.295 |
| 2-com | 22.63 | 77.37 |
| MetS | - | 43.945 |

**Section E:**

Standard error for transition rate estimate in Markov model by isolated states and times

| Time  Estimate | 0 | 3 | 6 | 9 | 12 | 15 | 18 | 21 |
| --- | --- | --- | --- | --- | --- | --- | --- | --- |
| NC | 0.0856 | 0.2273 | 0.3761 | 0.4123 | 0.4197 | 0.4211 | 0.4213 | 0.4214 |
| OB | 0.1447 | 0.2957 | 0.3993 | 0.4177 | 0.4208 | 0.4213 | 0.4214 | 0.4214 |
| HTN | 0.1580 | 0.2806 | 0.3845 | 0.4135 | 0.4198 | 0.4211 | 0.4213 | 0.4214 |
| DYS | 0.1307 | 0.2851 | 0.3867 | 0.4140 | 0.4200 | 0.4211 | 0.4213 | 0.4214 |
| HG | 0.1661 | 0.3243 | 0.4025 | 0.4177 | 0.4207 | 0.4212 | 0.4214 | 0.4214 |
| Standard error (SE) | 0.0142 | 0.0158 | 0.0049 | 0.0011 | 0.0002 | 0.0000 | 0.0000 | 0.0000 |

Standard error for transition rate estimate in Markov model by composite states and times

| Time  Estimate | 0 | 3 | 6 | 9 | 12 | 15 | 18 | 21 |
| --- | --- | --- | --- | --- | --- | --- | --- | --- |
| OB + HTN | 0.4109 | 0.4412 | 0.4236 | 0.4216 | 0.4214 | 0.4214 | 0.4214 | 0.4214 |
| OB + DYS | 0.2565 | 0.3787 | 0.4124 | 0.4197 | 0.4211 | 0.4213 | 0.4214 | 0.4214 |
| OB + HG | 0.3737 | 0.4409 | 0.4311 | 0.4236 | 0.4218 | 0.4215 | 0.4214 | 0.4214 |
| HTN + DYS | 0.3277 | 0.3929 | 0.4070 | 0.4176 | 0.4206 | 0.4212 | 0.4214 | 0.4214 |
| HTN + HG | 0.3846 | 0.4119 | 0.4158 | 0.4198 | 0.4210 | 0.4213 | 0.4214 | 0.4214 |
| DYS + HG | 0.2407 | 0.3541 | 0.4037 | 0.4174 | 0.4206 | 0.4212 | 0.4214 | 0.4214 |
| Standard error (SE) | 0.0287 | 0.0142 | 0.0042 | 0.0010 | 0.0002 | 0.0000 | 0.0000 | 0.0000 |

Standard error for transition rate estimate in MSD model by isolated states and times

| Time  Estimate | 0 | 3 | 6 | 9 | 12 | 15 | 18 | 21 |
| --- | --- | --- | --- | --- | --- | --- | --- | --- |
| NC | 0.0529 | 0.0465 | 0.0239 | 0.0509 | 0.0822 | 0.1422 | 0.2473 | 0.4310 |
| OB | 0.0546 | 0.2623 | 0.4618 | 0.7633 | 1.2790 | 2.1772 | 3.7479 | 6.5002 |
| HTN | 0.0569 | 0.1160 | -0.0441 | 0.1274 | 0.1288 | 0.2746 | 0.4555 | 0.8157 |
| DYS | 0.0636 | -0.0100 | 0.0100 | 0.0053 | 0.0133 | 0.0212 | 0.0377 | 0.0657 |
| HG | 0.0738 | 0.5145 | 0.4862 | 0.7389 | 1.2936 | 2.2529 | 3.9389 | 6.8896 |
| Standard error (SE) | 0.0038 | 0.0939 | 0.1176 | 0.1702 | 0.2973 | 0.5085 | 0.8840 | 1.5386 |

Standard error for transition rate estimate in MSD model by composite states and times

| Time  Estimate | 0 | 3 | 6 | 9 | 12 | 15 | 18 | 21 |
| --- | --- | --- | --- | --- | --- | --- | --- | --- |
| OB + HTN | 0.0253 | 0.1007 | 0.1965 | 0.3364 | 0.5917 | 1.0353 | 1.8156 | 3.1846 |
| OB + DYS | 0.0180 | 0.0146 | 0.0135 | 0.0207 | 0.0351 | 0.0612 | 0.1073 | 0.1885 |
| OB + HG | 0.0220 | 0.2708 | 0.6580 | 1.2215 | 2.1820 | 3.8433 | 6.7463 | 11.8306 |
| HTN + DYS | 0.0221 | -0.0018 | 0.0062 | 0.0046 | 0.0107 | 0.0175 | 0.0318 | 0.0556 |
| HTN + HG | 0.0239 | 0.1822 | 0.1911 | 0.2388 | 0.4540 | 0.7900 | 1.4041 | 2.4713 |
| DYS + HG | 0.0160 | 0.0139 | 0.0049 | 0.0156 | 0.0220 | 0.0416 | 0.0717 | 0.1268 |
| Standard error (SE) | 0.0014 | 0.0451 | 0.1029 | 0.1915 | 0.3420 | 0.6024 | 1.0573 | 1.8540 |
